# Does the environmental protection tax improve population health? Evidence from China’s quasi-natural experiment

**DOI:** 10.3389/fpubh.2026.1863545

**Published:** 2026-09-10

**Authors:** Junyao Tong, Bingnan Guo

**Affiliations:** School of Humanities and Social Sciences, Jiangsu University of Science and Technology, Zhenjiang, China

**Keywords:** difference-in-differences, environmental protection tax, investment in environmental pollution control, pollution reduction, population health

## Abstract

Health constitutes a fundamental component of human capital for high-quality economic development and an important indicator of social well-being. Using panel data for 31 Chinese provinces from 2010 to 2023, this study treats the implementation of the Environmental Protection Tax Law of the People’s Republic of China in 2018 as a quasi-natural experiment and employs a difference-in-differences model to systematically identify the effect of the environmental protection tax policy on population health. The results show that, first, the environmental protection tax policy significantly improves population health, and this finding remains robust after controlling for province fixed effects, year fixed effects, and a set of relevant covariates. Second, the baseline results are consistently supported by the parallel-trends test, placebo test, control for concurrent policies, replacement of the dependent variable, and propensity score matching difference-in-differences estimation. Third, the mechanism analysis indicates that pollution reduction and investment in environmental pollution control are important channels through which the environmental protection tax improves population health. Fourth, the regional heterogeneity analysis shows that the policy significantly improves population health in eastern and western China, whereas its effect in central China is positive but statistically insignificant, suggesting a certain degree of regional variation in policy effectiveness. This study extends the literature on the effects of environmental protection taxation from the perspective of population health and provides empirical evidence for improving the green taxation system and promoting coordination between ecological governance and the Healthy China initiative.

## 1 Introduction

Health constitutes an essential foundation for human capital accumulation, social welfare improvement, and high-quality economic development. Classical health economics regards health not only as an important consumption good that directly affects individual utility, but also as a form of capital stock that enhances labor productivity, extends effective working time, and improves long-term well-being. As research on health has continued to deepen, scholars have increasingly emphasized that health should not be viewed as a single and static outcome. Rather, it is a multidimensional condition jointly shaped by social determinants, resource endowments, public policies, and the external environment. Lines et al. (1) likewise stress that health measurement should move beyond reliance on a single indicator and adopt a more multidimensional and comprehensive perspective.

Alongside rapid industrialization, urbanization, and intensive energy consumption, environmental pollution has become a major external threat to population health. In recent years, a growing body of literature has examined health effects from the perspective of environmental governance rather than focusing solely on pollution exposure. Using China’s Two Control Zones policy as a quasi-natural experiment, Yang et al. (2) find that environmental regulation significantly improves public health. Xu et al. (3) further show that environmental regulation has a significant positive effect on health status, with improved air quality serving as an important transmission channel. Similarly, Xu et al. (4) report that stronger environmental regulation significantly reduces urban health risks. Beyond pollution control, improvements in the ecological environment are also closely associated with better health outcomes. Wang et al. (5), in their study of China’s green credit policy, demonstrate that green governance policies can generate health benefits by improving the ecological environment and optimizing resource allocation. Taken together, these studies reveal a robust relationship between environmental improvement and health promotion, thereby providing a direct basis for examining the health effects of the environmental protection tax.

Among environmental governance instruments, environmental taxation is widely regarded as an important institutional arrangement for correcting the negative externalities of pollution and promoting green transformation. Compared with the traditional pollution discharge fee system, the environmental protection tax is characterized by stronger legal authority, greater standardization, and more sustained regulatory constraints. Existing studies have primarily examined its policy effects in terms of pollution reduction, green innovation, corporate green transformation, and environmental performance. Li and Deng (6) show that the policy contributes positively to haze control, while Lin et al. (7) find that the environmental protection tax significantly strengthens pollution emission governance. Xue et al. (8) further demonstrate that the tax can, to some extent, reduce urban ozone pollution. Meanwhile, Deng et al. (9), Wang and Zhang (10), and Yu et al. (11) provide evidence on the micro-level behavioral effects of the environmental protection tax from the perspectives of green innovation in heavily polluting enterprises, environmental, social, and governance performance, and corporate green transformation, respectively. Overall, the existing literature has provided substantial evidence on the role of the environmental protection tax in pollution control and green transformation.

Nevertheless, the effects of environmental protection tax on population health and the underlying mechanisms remain insufficiently examined in the existing literature. First, existing studies have largely focused on the health effects of general environmental regulation or green policies, whereas relatively little attention has been paid to the direct relationship between the environmental protection tax and population health. Second, research on the environmental protection tax has mainly concentrated on pollution reduction, green innovation, and corporate transformation, while its health and welfare effects have not been adequately explored. Third, the specific channels through which the environmental protection tax affects population health, as well as the regional heterogeneity of these effects, have yet to be systematically investigated.

Against this background, this study uses provincial-level panel data for China from 2010 to 2023 to systematically examine the effect of the environmental protection tax on population health in the institutional context of its implementation in 2018. Specifically, this study first constructs a composite population health index using the entropy weight method, based on indicators including the number of beds in medical institutions, the number of health technicians, the mortality rate, and the hospital bed utilization rate. It then employs a difference-in-differences model to identify the net effect of the environmental protection tax and conducts a series of robustness checks, including a parallel-trends test, a placebo test, controls for concurrent policies, replacement of the dependent variable, and propensity score matching combined with difference-in-differences estimation. Building on these analyses, this study further investigates two potential channels pollution reduction and investment in environmental pollution control and divides the sample into eastern, central, and western regions to examine the regional heterogeneity of the policy’s health effects.

The marginal contributions of this study are threefold. First, it extends research on the policy effects of the environmental protection tax from the perspective of population health. Second, it constructs a multidimensional composite health index using the entropy weight method, thereby improving the comprehensiveness and objectivity of health measurement. Third, it investigates the potential channels through which the environmental protection tax affects population health from the perspectives of pollution reduction and investment in environmental pollution control, while also identifying the regional heterogeneity of the policy effects. By examining whether regional differences in policy effects are associated with differences in health outcomes, this study further evaluates the effectiveness of the environmental protection tax and provides empirical evidence for improving the environmental taxation system and advancing the Healthy China initiative.

## 2 Theoretical analysis and research hypotheses

Population health should not be understood merely as the absence of disease; rather, it is a multidimensional outcome jointly shaped by environmental quality, the provision of public resources, and institutional governance. From this perspective, the health effects of the environmental protection tax cannot be interpreted solely in terms of the tax burden imposed on firms. Instead, they should be examined within a transmission chain of “policy constraints-corporate responses-environmental improvement-health benefits.” As a typical market-based environmental regulation instrument, the environmental protection tax primarily operates by increasing the economic cost of pollution emissions and thereby reshaping firms’ trade-offs among emissions, pollution-control investment, and production returns. Goulder (12) argues that environmental taxation not only corrects environmental externalities but may also generate additional efficiency gains by encouraging the reallocation of resources. Consistent with this theoretical logic, recent empirical evidence shows that environmental regulation can significantly affect firms’ production patterns and environmental governance behavior. Based on evidence from China’s cement industry, Jia and Luo (13) find that environmental regulation significantly improves population health outcomes, with tighter emission constraints on highly polluting industries and stronger environmental governance practices serving as important underlying conditions. Similarly, in their study of pollution-control policies in China’s “2 + 26″ cities, Xie et al. (14) show that stringent environmental regulation can produce substantial improvements in air quality and corresponding health benefits, indicating a clear environmental pathway through which environmental governance policies affect population health. Therefore, although the environmental protection tax directly targets the corporate sector, it may improve regional environmental quality by strengthening firms’ pollution-control responsibilities, promoting emission reduction, and facilitating green adjustment.

More specifically, reductions in corporate pollution emissions and improvements in environmental quality may ultimately affect health by lowering residents’ exposure to pollution, reducing the incidence of disease, and alleviating pressure from medical expenditures. Pope et al. (15) provide early evidence that long-term exposure to fine particulate matter significantly increases the risk of mortality from cardiopulmonary disease and lung cancer. In the Chinese context, Ebenstein et al. (16) further demonstrate that sustained exposure to severe pollution leads to substantial losses in life expectancy. More recent studies have provided direct evidence linking environmental improvement to health and welfare gains. The systematic review by Gascon et al. (17) documents a relatively stable positive association between access to green space and favorable health outcomes, further suggesting that improvements in the ecological environment can generate observable health benefits. Meanwhile, Jin and Li (18) find that air pollution increases healthcare utilization and medical expenditure burdens. Accordingly, when the environmental protection tax induces firms to reduce pollution emissions and improve regional environmental quality, residents’ exposure-related health risks are likely to decline, thereby improving population health. Based on the above analysis, this study proposes the following hypothesis:

*H1*: The environmental protection tax policy significantly improves population health.

Pollutant emissions constitute an important source of environmental health risk. Air pollutants such as sulfur dioxide increase the risk of respiratory and cardiovascular diseases and impose additional healthcare burdens. By increasing the economic cost of pollution emissions, the environmental protection tax reduces the marginal returns from continued polluting activities and creates sustained incentives for emission reduction. First, the environmental protection tax reinforces the polluter-pays principle, thereby encouraging firms to reduce highly polluting production activities, improve production processes, and adopt cleaner technologies. Second, closer coordination between tax administration and pollution monitoring can enhance environmental supervision by local governments. These mechanisms may jointly contribute to reductions in sulfur dioxide and other pollutant emissions, thereby lowering residents’ exposure to air pollution and the associated health risks. Accordingly, this study proposes the following hypothesis:

*H2*: The environmental protection tax policy improves population health by reducing pollutant emissions.

In addition to directly reducing pollution emissions, the environmental protection tax may also affect population health by promoting investment in pollution control. As the tax cost of continued emissions and the risk of environmental violations increase, firms may invest more in pollution-control facilities, cleaner production technologies, production-process upgrading, and end-of-pipe treatment equipment in order to reduce long-term operating costs. Increased investment in pollution control can enhance firms’ pollution-treatment capacity, facilitate the upgrading of production equipment and treatment facilities, and reduce the discharge of pollutants into the external environment. At the same time, implementation of the environmental protection tax may increase the attention paid by both local governments and firms to environmental governance performance and promote the allocation of governance resources toward key polluting industries and priority pollution-control areas. As pollution-control investment and environmental governance capacity improve, regional environmental quality is likely to improve further, while residents’ pollution exposure and health risks decline accordingly. Based on this reasoning, this study proposes the following hypothesis:

*H3*: The environmental protection tax policy improves population health by increasing investment in pollution control.

The effects of the environmental protection tax on population health may also depend on regional development conditions and environmental governance capacity. Eastern, central, and western China differ substantially in terms of economic development, industrial structure, environmental governance capacity, and the provision of public services. Eastern China generally has a stronger economic foundation, more effective environmental regulation, and greater capacity for the adoption of green technologies. Central China is undergoing industrial restructuring and economic transformation and remains relatively dependent on traditional industries. Western China, by contrast, has more fragile ecological conditions and greater potential for marginal improvements through pollution control. Consequently, both the implementation effects of the environmental protection tax and the extent to which those effects are translated into health benefits may vary across regions. Based on the above analysis, this study proposes the following hypothesis:

*H4*: The effect of the environmental protection tax policy on population health exhibits regional heterogeneity.

## 3 Research design

### 3.1 Model specification

#### 3.1.1 Baseline difference-in-differences model

This study treats the implementation of the Environmental Protection Tax Law in 2018 as an exogenous policy shock and employs a difference-in-differences model to identify the effect of the environmental protection tax policy on population health. The principal advantage of the difference-in-differences approach is that it can estimate the net effect of a policy intervention by comparing changes in the treatment and control groups before and after policy implementation. Accordingly, this method is well suited to the objective of evaluating the health effects of the environmental protection tax policy.

From an institutional perspective, the Environmental Protection Tax Law came into force in 2018, marking China’s formal transition from the pollution discharge fee system to the environmental protection tax system. This institutional reform was supported by a clear legal framework and implemented at a uniform policy date, thereby providing favorable quasi-natural experimental conditions for identifying the policy effects of the environmental protection tax. In addition, existing empirical studies on the environmental protection tax commonly regard 2018 as the key policy intervention year and conduct policy evaluations accordingly. Based on this institutional setting, this study specifies a difference-in-differences model to examine the net effect of the environmental protection tax policy on population health. Accordingly, the baseline difference-in-differences model is specified in Equation 1 as follows:


$$\mathrm{ch}i_{\mathrm{it}}=\alpha +\mathrm{\beta DI}D_{\mathrm{it}}+\gamma X_{\mathrm{it}}+\mu_{i}+\lambda_{t}+\varepsilon_{\mathrm{it}}$$ (1)

Where i and t denote province and year, respectively. $\mathrm{ch}i_{\mathrm{it}}$ is the dependent variable and represents the composite population health index of province i in year t. $\mathrm{DI}D_{\mathrm{it}}$ is the core explanatory variable and denotes the difference-in-differences term for the environmental protection tax policy. $X_{\mathrm{it}}$ is a vector of control variables, including per capita park green area, the green coverage rate of built-up areas, gross domestic product, local fiscal expenditure on healthcare, and the number of health institutions. $\mu_{i}$ denotes province fixed effects, which control for time-invariant province-specific characteristics. $\lambda_{t}$ denotes year fixed effects, which capture annual shocks common to all provinces. $\varepsilon_{\mathrm{it}}$ is the idiosyncratic error term.

The coefficient of primary interest is β . A significantly positive estimate of β indicates that the environmental protection tax policy significantly increases the composite population health index and therefore improves population health. An insignificant estimate, by contrast, suggests that the policy effect on population health is not statistically distinguishable from zero.

#### 3.1.2 Mechanism analysis model

The baseline regression model can identify whether the environmental protection tax policy significantly affects population health, but it does not reveal the specific channels through which this effect operates. To further investigate the potential transmission mechanisms linking the environmental protection tax to population health, this study examines whether the policy significantly affects the relevant mechanism variables, thereby providing channel-specific empirical evidence for its health effects. Based on the preceding theoretical analysis and the empirical framework of this study, the environmental protection tax is expected to influence population health primarily through two channels: pollution reduction and investment in environmental pollution control.

On the one hand, by increasing the cost of pollution emissions and strengthening firms’ incentives to reduce emissions, the environmental protection tax encourages firms to adjust their production methods, adopt cleaner production technologies, and reduce pollutant emissions. Sulfur dioxide is a representative air pollutant, and reductions in its emissions can help decrease residents’ exposure to air pollution and the associated health risks. Therefore, if the environmental protection tax significantly reduces sulfur dioxide emissions, this would provide evidence that pollution reduction constitutes an important channel through which the policy improves population health.

On the other hand, implementation of the environmental protection tax increases the economic cost of continued pollution emissions, requiring firms to reassess the trade-offs among pollutant emissions, tax liabilities, and pollution-control expenditures. To reduce long-term emission costs and the risk of environmental violations, firms may increase investment in pollution-control facilities, production-process upgrading, and end-of-pipe treatment. Greater investment in industrial pollution control can enhance pollution-treatment capacity, reduce pollutant emissions, and improve regional environmental quality, thereby lowering the environmental health risks faced by residents. Accordingly, investment in environmental pollution control may constitute another important channel through which the environmental protection tax improves population health.

To identify these channels, this study uses sulfur dioxide emissions and completed investment in industrial pollution control as the mechanism variables and specifies the mechanism analysis model in Equation 2 as follows:


$$M_{\mathrm{it}}=\alpha +\mathrm{\beta DI}D_{\mathrm{it}}+\gamma X_{\mathrm{it}}+\mu_{i}+\lambda_{t}+\varepsilon_{\mathrm{it}}$$ (2)

Where *i* and *t* denote province and year, respectively. $M_{\mathrm{it}}$ represents the mechanism variable, measured by sulfur dioxide emissions and completed investment in industrial pollution control, respectively. $\mathrm{DI}D_{\mathrm{it}}$ denotes the difference-in-differences term for the environmental protection tax policy. $X_{\mathrm{it}}$ is a vector of control variables, including per capita park green area, the green coverage rate of built-up areas, gross domestic product, local fiscal expenditure on healthcare, and the number of health institutions. γ is the corresponding vector of coefficients to be estimated. $\mu_{i}$ and $\lambda_{t}$ denote province fixed effects and year fixed effects, respectively, while $\varepsilon_{\mathrm{it}}$ is the idiosyncratic error term. Standard errors are clustered at the provincial level.

When $M_{\mathrm{it}}$ is measured by sulfur dioxide emissions, a significantly negative estimate of the coefficient on $\alpha_{1}$ indicates that the environmental protection tax policy effectively reduces sulfur dioxide emissions, thereby providing empirical support for the pollution-reduction channel. When $M_{\mathrm{it}}$ is measured by completed investment in industrial pollution control, a significantly positive estimate of the coefficient on $\alpha_{1}$ indicates that the policy promotes investment in industrial pollution control, thereby providing empirical support for the environmental-governance-investment channel.

Through this model, the study further examines whether the environmental protection tax policy significantly affects pollutant emissions and investment in environmental pollution control. The results therefore provide channel-specific empirical evidence on whether the policy improves population health through pollution reduction and strengthened environmental governance.

### 3.2 Variable selection

#### 3.2.1 Dependent variable: composite population health index

The dependent variable in this study is the composite population health index ( *chi* ). Population health is an important indicator of regional well-being and the quality of social development and is inherently multidimensional. It is reflected not only in population health outcomes but also in the availability of medical resources and the utilization of healthcare services. Because a single indicator cannot comprehensively capture population health, this study draws on the World Health Organization’s conceptualization of health and, subject to the availability of provincial panel data, selects indicators from three dimensions: medical resource provision, healthcare service capacity, and health outcomes. The composite population health index is then constructed using the entropy weight method.

The entropy weight method assigns objective weights according to the degree of dispersion of each indicator. Indicators with greater variation contain more information and therefore receive larger weights in the composite evaluation. Compared with subjective weighting methods, the entropy weight method can reduce bias arising from discretionary judgment and improve the objectivity and scientific rigor of composite index measurement.

Specifically, *mib* denotes the number of beds in medical institutions, which primarily reflects the scale of regional healthcare infrastructure and serves as an important indicator of medical resource allocation capacity. Htp denotes the number of health technical personnel, reflecting the availability of human resources for healthcare services; a larger number generally implies greater accessibility and effectiveness of medical services for residents. *Mor* denotes the mortality rate, which is an important indicator of population health outcomes and the regional burden of disease. A higher mortality rate indicates poorer population health. *Hbu* denotes the hospital bed utilization rate, which reflects the actual utilization of medical resources and the operational performance of healthcare services and, to some extent, captures the service capacity and resource allocation efficiency of medical institutions.

It should be noted that *mor* is a negative indicator, meaning that a higher value represents a lower level of population health. It is therefore reverse-standardized before the index is constructed. The remaining indicators are positive indicators, for which higher values generally indicate better health-related conditions. After standardization, all indicators are transformed so that higher values consistently represent better population health.

For positive indicators, the standardization procedure is specified in Equation 3 as follows:


$$Z_{\mathrm{ijt}}=\frac{X_{\mathrm{ijt}}-\min(X_{\mathrm{jt}})}{\max(X_{\mathrm{jt}})-\min(X_{\mathrm{jt}})}$$ (3)

For negative indicators, reverse standardization is performed according to Equation 4 as follows:


$$Z_{\mathrm{ijt}}=\frac{\max(X_{\mathrm{jt}})-X_{\mathrm{ijt}}}{\max(X_{\mathrm{jt}})-\min(X_{\mathrm{jt}})}$$ (4)

Where $\;X_{\mathrm{ijt}}\;$ denotes the original value of indicator *j* for province *i* in year *t* , and $Z_{\mathrm{ijt}}\;$ denotes the corresponding standardized value. Reverse standardization of the mortality rate ensures directional consistency across all components of the composite index, such that a higher index value consistently indicates a better level of population health.

After standardization, the proportion of indicator *j* for province *i* in year *t* is calculated according to Equation 5:


$$P_{\mathrm{ijt}}=\frac{Z_{\mathrm{ijt}}}{\sum_{i=1}^{n}Z_{\mathrm{ijt}}}$$ (5)

The information entropy of indicator *j* is then calculated according to Equation 6:


$$e_{\mathrm{jt}}=-k\sum_{i=1}^{n}P_{\mathrm{ijt}}\ln P_{\mathrm{ijt}}$$ (6)

Where the constant *k* is defined in Equation 7:


$$k=\frac{1}{\mathrm{lnn}}$$ (7)

denotes the number of observations in the sample. The degree of diversification is then calculated according to Equation 8:


$$d_{\mathrm{jt}}=1-e_{\mathrm{jt}}$$ (8)

The weight assigned to each indicator is then determined accordingly to Equation 9:


$$w_{\mathrm{jt}}=\frac{d_{\mathrm{jt}}}{\sum_{j=1}^{m}d_{\mathrm{jt}}}$$ (9)

Finally, the composite population health index is calculated according to Equation 10 as follows:


$$\mathrm{ch}i_{\mathrm{it}}=\sum_{j=1}^{m}w_{\mathrm{jt}}Z_{\mathrm{ijt}}$$ (10)

Where $\mathrm{ch}i_{\mathrm{it}}$ denotes the composite population health index for province *i* in year *t* . A higher value of the index indicates a higher level of population health in that province. Aggregating multiple health-related indicators into a single measure using the entropy weight method enables a more comprehensive and objective assessment of regional population health.

#### 3.2.2 Explanatory variable

The core explanatory variable in this study is the environmental protection tax policy variable. On January 1, 2018, the Environmental Protection Tax Law of the People’s Republic of China officially came into effect, marking the formal transition from the pollution discharge fee system to the environmental protection tax system in China. Given that the Environmental Protection Tax Law has been implemented nationwide since 2018, this study defines 2018 and subsequent years as the post-policy period and constructs the time dummy variable $\mathrm{Pos}t_{t}$ . Specifically, $\mathrm{Pos}t_{t}$ *=1* for 2018 and all subsequent years, and $\mathrm{Pos}t_{t}$ *=0* otherwise.

Following the implementation of the Environmental Protection Tax Law, 12 provinces-Guizhou, Hainan, Guangxi, Shanxi, Beijing, Hebei, Henan, Chongqing, Jiangsu, Shandong, Hunan, and Sichuan-raised their applicable environmental protection tax rates. Accordingly, this study constructs the treatment-group indicator $\text{Trea}t_{i}$ and further generates the difference-in-differences interaction term $\mathrm{DI}D_{\mathrm{it}}=\text{Trea}t_{i}\times \mathrm{Pos}t_{t}$ . The interaction term $\mathrm{DI}D_{\mathrm{it}}$ captures the net policy effect arising from the implementation of the environmental protection tax. Its estimated coefficient indicates the direction and magnitude of the policy’s effect on the composite population health index and therefore constitutes the key variable for identifying the policy effect in this study.

#### 3.2.3 Control variables

Drawing on the existing literature in health economics, environmental health, and healthcare services, and considering the continuity and availability of provincial panel data, this study selects a set of factors that may affect population health as control variables to mitigate omitted-variable bias and improve the reliability of the estimation results.

Specifically, per capita park green area ( *pga* ) is measured as the ratio of the total area of urban parks and green spaces to the permanent resident population. Adequate access to green space may reduce health risks by improving air quality and promoting physical activity. Twohig-Bennett and Jones (19) documented robust associations between exposure to natural environments and better physical and mental health, as well as a lower risk of mortality, suggesting that the provision of green space constitutes an important environmental foundation for population health.

The green coverage rate of built-up areas ( *gcr* ) is measured as the proportion of green-covered land in the total built-up area and captures the level of regional greening and the ecological carrying capacity of the local environment. Gross domestic product ( *gdp* ) is measured by regional GDP and reflects the level of regional economic development. Economic development provides the material basis for public infrastructure, healthcare provision, and environmental governance, while also affecting health through industrial structure, resource allocation, and residents’ living conditions.

Local fiscal expenditure on healthcare ( *fhe* ) is measured by the total annual provincial government expenditure on healthcare and reflects the intensity of government investment in the health sector. Adequate fiscal support can expand healthcare supply and strengthen the public health system. Self and Grabowski (20) argued that public health expenditure is an important institutional condition for improving overall health, and that increased fiscal investment generally enhances the capacity of public health services and the level of health protection.

The number of health institutions ( *hin* ) covers all types of healthcare facilities and is used to measure the scale of healthcare service provision. The availability and spatial distribution of such institutions directly affect access to healthcare and support residents’ medical treatment and health management. Basu et al. (21) found that greater availability of primary care resources was significantly associated with lower population mortality, indicating that improved access to healthcare services plays an important role in enhancing health outcomes.

Based on the above considerations, *pga* , *gcr* , *gdp* , *fhe* , and *hin* are included in the baseline regression model. Province fixed effects and year fixed effects are also incorporated to account, respectively, for time-invariant regional characteristics and common macroeconomic shocks across years.

#### 3.2.4 Data sources

Considering data availability, completeness, and accuracy, this study employs a balanced panel dataset covering 31 provincial-level administrative regions in China from 2010 to 2023, excluding Hong Kong, Macao, and Taiwan. The data are obtained from the EPS Global Statistical Data, the China Health Statistical Yearbook, the National Bureau of Statistics of China, provincial and municipal statistical yearbooks, and reports on the development of health services. The dataset contains only one missing observation, which is imputed using linear interpolation. Descriptive statistics for the variables used in this study are reported in Table 1.

**Table 1:** Descriptive statistics of main variables.

Variables	Obs	Mean	SD	Min	Max
chi	434	0.4349	0.1476	0.1647	0.8881
DID	434	0.1659	0.3724	0	1
pga	434	2.5590	0.2296	1.7544	3.1284
gcr	434	4.0608	0.5413	1.8060	6.3548
gdp	434	9.8002	1.0090	6.2314	11.8180
fhe	434	5.8110	0.7852	3.4977	7.6412
hin	434	10.1319	0.9542	8.3260	12.9596

## 4 Empirical results and analysis

### 4.1 Measurement results of population health levels

**Figure 1:**
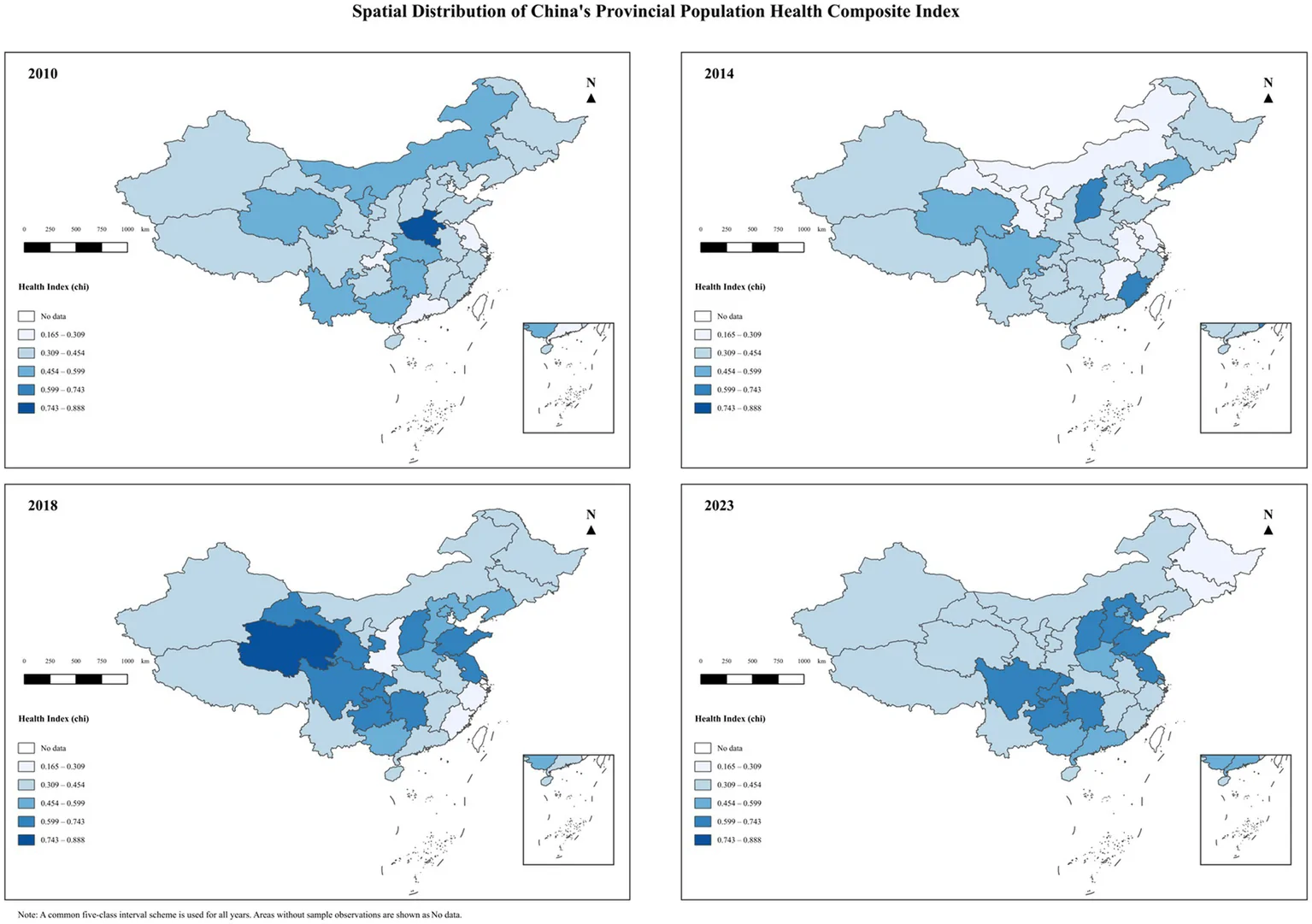
Trends in population health levels (2010–2023).

This study employs the entropy weight method to estimate population health levels across Chinese provinces and maps their spatial distributions in 2010, 2014, 2018, and 2023, as shown in Figure 1. From a temporal perspective, population health in China exhibits an overall upward trend, although the pace and pattern of change vary considerably across regions. Against the backdrop of the implementation of the environmental protection tax policy, population health generally improved across provinces. The improvement was particularly pronounced in some eastern and central regions, while western regions also experienced a gradual increase in population health. Overall, regional disparities in population health persisted, but the spatial distribution pattern underwent substantial dynamic changes over the sample period.

### 4.2 Analysis of the baseline regression results

Table 2 reports the baseline regression results for the effect of the environmental protection tax policy on population health. Column (1) includes only province fixed effects. The estimated coefficient on DID is 0.1824 and is statistically significant at the 1% level, indicating that population health improved significantly following the implementation of the environmental protection tax policy. After the control variables are added in Column (2), the coefficient on DID decreases slightly to 0.1704 but remains statistically significant at the 1% level. Column (3) further incorporates year fixed effects, yielding a positive DID coefficient of 0.1571, which remains significant at the 1% level.

**Table 2:** Baseline regression results.

Variables	(1)	(2)	(3)
DID	0.1824*** (0.0249)	0.1704*** (0.0331)	0.1571*** (0.0342)
Controls	No	Yes	Yes
Year FE	No	No	Yes
Province FE	Yes	Yes	Yes
Obs	434	434	434
*R*-squared	0.1732	0.1838	0.2208

Standard errors in parentheses, *** *p* < 0.01, ** *p* < 0.05, * *p* < 0.1.

These results demonstrate that the beneficial effect of the environmental protection tax policy on population health remains robust regardless of whether the control variables and year fixed effects are included. Notably, although the estimated coefficient decreases moderately as additional controls and year fixed effects are introduced, its sign and statistical significance remain unchanged. This finding suggests that the estimated health effect of the environmental protection tax policy is unlikely to be driven by time-invariant provincial characteristics or common annual shocks, thereby providing robust support for the policy’s positive effect on population health.

### 4.3 Robustness tests

#### 4.3.1 Parallel trends test

**Figure 2:**
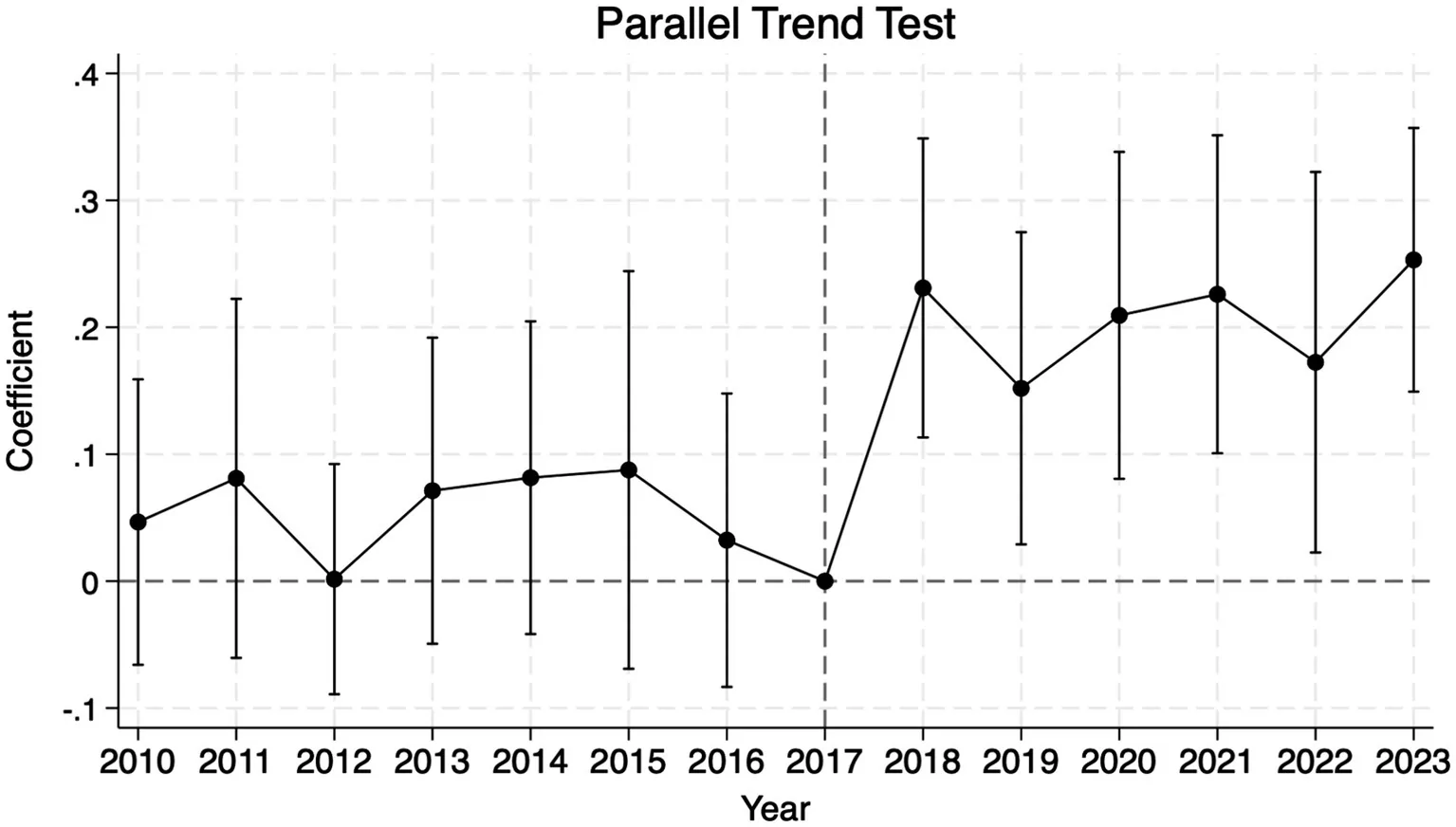
Parallel trend test.

Figure 2 presents the results of the parallel trends test for the environmental protection tax policy. Prior to policy implementation, the estimated coefficients for 2010–2017 fluctuate around zero, and their corresponding confidence intervals all include zero. No statistically significant systematic differences in pre-policy trends are observed, indicating that the treatment and control groups were broadly comparable before the policy shock and that the parallel trends assumption is reasonably satisfied.

Following policy implementation, the estimated coefficients for 2018 and subsequent years are generally positive and noticeably larger than those in the pre-policy period, suggesting that the environmental protection tax policy exerted a significant positive effect on population health. Moreover, the policy effect persists in subsequent years and exhibits a certain degree of dynamic accumulation. This finding indicates that the beneficial effect of the environmental protection tax on population health is not attributable to a short-term random fluctuation but is relatively persistent over time. Overall, the parallel trends test provides further support for the reliability of the baseline regression results.

#### 4.3.2 Placebo test

To rule out interference from non-policy factors and further assess the validity of the causal identification of the effect of the environmental protection tax policy on population health, this study conducts a placebo test. Specifically, while holding the sample structure and baseline model specification constant, treatment provinces are randomly assigned and a placebo policy interaction term is constructed. This procedure is repeated 500 times to examine the distribution of the estimated coefficients on the core explanatory variable under fictitious policy shocks.

**Figure 3:**
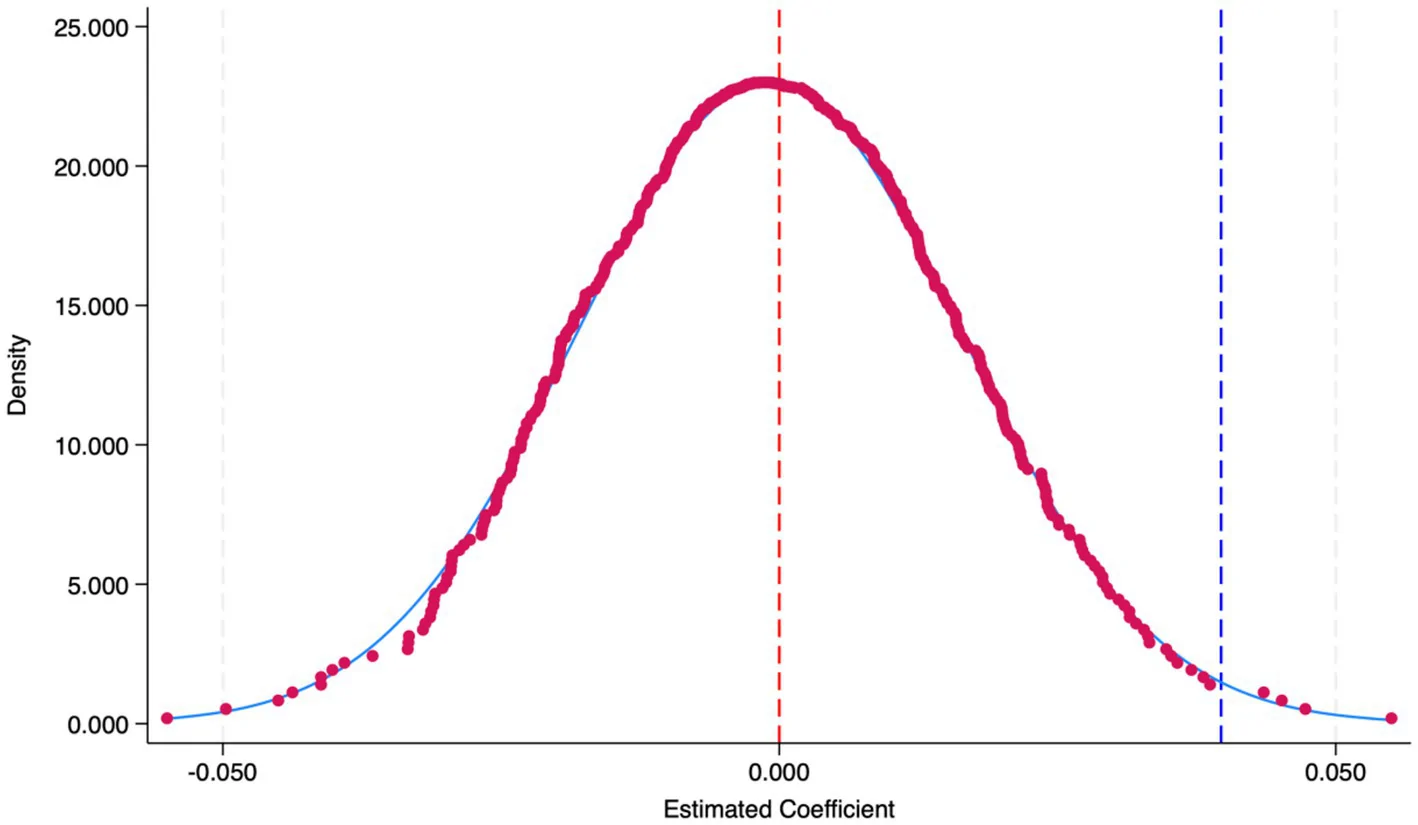
Placebo test.

Figure 3 presents the placebo test results. The coefficients obtained from the random permutations are concentrated around zero and exhibit a broadly symmetric distribution, indicating that the placebo policy variable has no systematic effect on population health in the absence of an actual policy shock. By contrast, the coefficient estimated in the baseline regression deviates markedly from the center of the placebo distribution and lies in its right tail. This finding suggests that the estimated effect of the environmental protection tax policy is unlikely to be driven by random disturbances or other unobserved factors and is therefore highly robust. Overall, the placebo test provides further support for the reliability of the baseline regression findings.

#### 4.3.3 Controlling for the effects of other policies

To rule out potential interference from other environmental policies implemented during the same period, this study conducts an additional robustness test by controlling for the effects of concurrent policies. The central environmental protection inspection program was implemented in successive batches during the sample period and may have affected population health by strengthening local environmental governance and improving environmental quality, thereby potentially confounding the identification of the net effect of the environmental protection tax policy. Accordingly, this study constructs a central environmental protection inspection policy variable based on the year in which each province was first subject to a central environmental protection inspection and includes this variable together with the environmental protection tax policy variable in the baseline regression model.

Table 3 shows that, before controlling for the Central Environmental Protection Inspection policy, the estimated coefficient on the Environmental Protection Tax policy variable is 0.1571 and is significantly positive at the 1% level. After additionally controlling for the Central Environmental Protection Inspection policy, the estimated coefficient on DID is 0.1582, which remains significantly positive at the 1% level and is highly consistent with the baseline regression result. The two specifications both contain 434 observations, while the R-squared increases slightly from 0.3474 to 0.3486. These results indicate that the positive effect of the Environmental Protection Tax on population health remains statistically significant and stable after accounting for the potential influence of the concurrent Central Environmental Protection Inspection policy. This suggests that the baseline findings are not driven by this contemporaneous environmental policy and further confirms the robustness of the empirical results.

**Table 3:** Robustness check results.

Variables	(1) Controlling for CEPI	(2) Mortality rate	(3) PSM-DID
DID	0.1582*** (0.0340)	−0.0463* (0.0232)	0.1808*** (0.0393)
DID1	0.0384 (0.0384)		
Controls	Yes	Yes	Yes
Year FE	Yes	Yes	Yes
Province FE	Yes	Yes	Yes
Obs	434	434	266
*R*-squared	0.3486	0.8551	0.4607

Standard errors in parentheses, *** *p* < 0.01, ** *p* < 0.05, * *p* < 0.1.

#### 4.3.4 Replacing the dependent variable

To examine whether the baseline findings depend on the specific measurement of population health, this study uses the mortality rate as an alternative dependent variable. Column (2) of Table 3 shows that, after replacing the dependent variable with the mortality rate, the estimated coefficient on the environmental protection tax policy variable is −0.0463 and is statistically significant at the 10% level. This result indicates that the environmental protection tax policy significantly reduces the mortality rate. Because mortality is a negative health indicator, this finding is consistent with the baseline result that the environmental protection tax policy significantly improves population health. Overall, after the dependent variable is replaced, both the sign and statistical significance of the core explanatory variable support the baseline findings, confirming the robustness of the empirical results.

#### 4.3.5 PSM-DID test

To mitigate potential systematic differences between the treatment and control groups prior to policy implementation, as well as the resulting sample selection bias, this study further employs a combination of propensity score matching and difference-in-differences as a robustness check.

For the estimation of propensity scores, per capita park green area ( *pga* ), the green coverage rate of built-up areas ( *gcr* ), gross domestic product ( *gdp* ), local fiscal expenditure on healthcare ( *fhe* ), and the number of healthcare institutions ( *hin* ) are selected as matching covariates. To avoid potential contamination from post-treatment variables that may themselves be affected by the Environmental Protection Tax policy, the provincial averages of these covariates over the pre-policy period from 2010 to 2017 are used to estimate the propensity scores. Specifically, a Logit model is employed to estimate the probability of each province being assigned to the treatment group. Subsequently, 1:1 nearest-neighbor matching with replacement is conducted within the region of common support, with the caliper set at 0.05.

**Figure 4:**
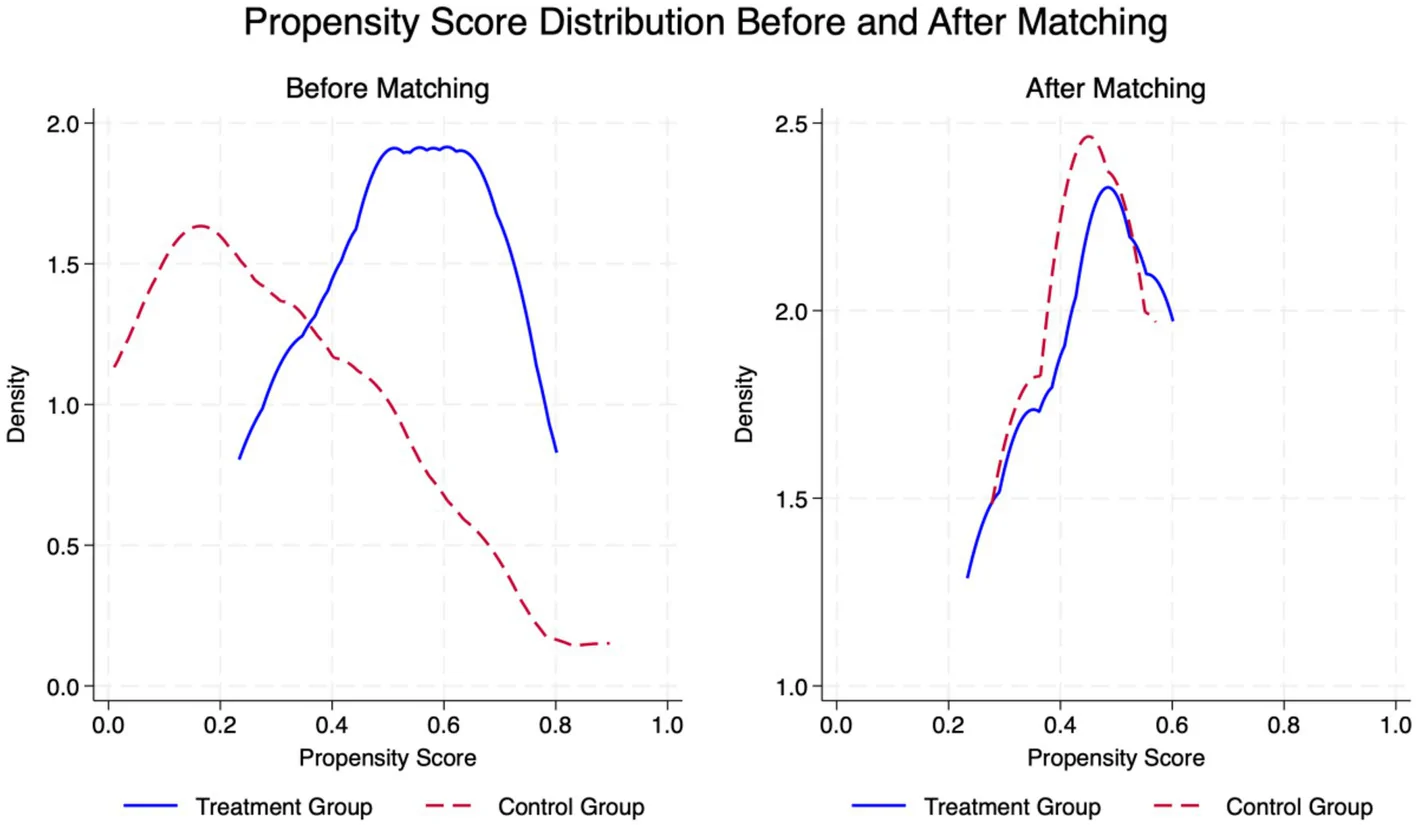
Distribution of propensity scores for the matched sample.

Figure 4 presents the kernel density distributions of the propensity scores for the treatment and control groups before and after matching. Prior to matching, the two groups exhibit noticeable differences in their propensity score distributions. After matching, however, the degree of overlap between the kernel density curves increases substantially within the common support region, and the propensity scores of the two groups become concentrated within similar ranges. These results indicate that the common support condition is broadly satisfied and that propensity score matching effectively improves the overall comparability between the treatment and control groups.

On this basis, the successfully matched provinces are merged back into the full panel dataset covering the period from 2010 to 2023, and the DID model is re-estimated using the matched sample. As reported in Column (3) of Table 3, the estimated coefficient of the Environmental Protection Tax policy variable, DID, is 0.1808 and is significantly positive at the 1% level. Both the direction and statistical significance of the estimate are consistent with the baseline regression results. Although the number of observations decreases from 434 to 266 after matching, the positive effect of the Environmental Protection Tax on population health remains statistically significant. These findings indicate that, after accounting for observable differences between the treatment and control groups through propensity score matching, the baseline results remain robust.

### 4.4 Mechanism analysis

To further identify the potential channels through which the environmental protection tax affects population health, this study follows the two-step mechanism-testing approach proposed by Jiang Ting (22). Based on the preceding theoretical analysis, the mechanisms are examined from two dimensions: pollution reduction and investment in environmental governance. These two mechanism variables are selected because the environmental protection tax, as a market-based environmental regulation instrument, may operate through two principal channels. First, by increasing the cost of pollutant emissions and strengthening firms’ incentives to reduce emissions, the policy may induce enterprises to cut pollutant discharges, thereby lowering residents’ exposure to pollution and improving health outcomes. Second, by tightening environmental governance constraints and increasing investment in pollution control, the policy may encourage firms to upgrade production processes and pollution-control facilities, ultimately improving regional environmental quality and population health.

Table 4 reports the mechanism analysis results. In Column (1), sulfur dioxide emissions ( *soe* ) are used as the dependent variable. The estimated coefficient on DID is −0.5510 and is statistically significant at the 10% level, indicating that the environmental protection tax policy reduces sulfur dioxide emissions to some extent. This finding provides empirical support for the pollution-reduction channel. In Column (2), investment in industrial pollution control ( *pci* ) is used as the dependent variable. The estimated coefficient on DID is 0.7353 and is significantly positive at the 5% level, suggesting that the environmental protection tax policy significantly increases investment in industrial pollution control and thereby provides further support for the environmental-governance-investment channel.

**Table 4:** Mechanism analysis results.

Variables	(1) *soe*	(2) *pci*
DID	−0.5510* (0.3088)	0.7353** (0.3565)
Controls	Yes	Yes
Year FE	Yes	Yes
Province FE	Yes	Yes
Obs	434	434

Standard errors in parentheses, *** *p* < 0.01, ** *p* < 0.05, * *p* < 0.1.

Overall, the mechanism analysis indicates that the environmental protection tax policy not only significantly improves population health but also operates through two channels: reducing sulfur dioxide emissions and increasing investment in pollution control. Specifically, the pollution-reduction mechanism is supported at the 10% significance level, whereas the environmental-governance-investment mechanism is supported at the 5% significance level.

### 4.5 Regional heterogeneity

The preceding analysis demonstrates that the environmental protection tax policy significantly improves population health. To further examine whether the policy effect varies across regions, the sample is divided into eastern, central, and western regions, and separate regressions are estimated for each subsample. As shown in Table 5, the estimated policy coefficients for the eastern and western regions are 0.1571 and 0.2004, respectively, both of which are significantly positive, indicating that the environmental protection tax policy effectively improves population health in these two regions. For the central region, the estimated coefficient is 0.3465; although positive, it is not statistically significant.

**Table 5:** Regional heterogeneity analysis results.

Variables	(1) Eastern region	(2) Central region	(3) Western region
DID	0.1571** (0.0507)	0.3465 (0.1965)	0.2004*** (0.0416)
Controls	Yes	Yes	Yes
Year FE	Yes	Yes	Yes
Province FE	Yes	Yes	Yes
Obs	154	112	168
*R*-squared	0.3780	0.4847	0.4632

Standard errors in parentheses, *** *p* < 0.01, ** *p* < 0.05, * *p* < 0.1.

Several factors may account for these regional differences. The eastern region generally has a stronger economic foundation and greater environmental governance capacity, enabling it to translate regulatory constraints into health benefits more effectively. The western region is characterized by relatively fragile ecological conditions, which may make the marginal health benefits of improvements in pollution control more pronounced. By contrast, the central region remains in a period of industrial restructuring, and the policy effect may be constrained by continued dependence on traditional industries and regional disparities in environmental governance capacity.

## 5 Conclusions and policy implications

Using panel data for 31 Chinese provinces from 2010 to 2023, this study treats the formal implementation of the Environmental Protection Tax Law in 2018 as a policy shock and employs a difference-in-differences model to empirically examine the effect of the environmental protection tax policy on population health. The main findings are as follows. First, the environmental protection tax policy significantly improves population health. Second, the policy may affect population health primarily through two channels: pollution reduction and increased investment in environmental governance. Third, the regional heterogeneity analysis shows that the policy significantly promotes population health in the eastern and western regions. Although the estimated coefficient for the central region is positive, it is not statistically significant, indicating a certain degree of regional variation in the health effects of the policy.

Based on these findings and China’s institutional context, this study proposes the following policy implications and recommendations to improve the environmental governance system and strengthen the health-promoting effects of the environmental protection tax.

First, the synergistic role of the environmental protection tax in advancing the Healthy China initiative should be further strengthened. The environmental protection tax not only contributes to pollution control and green transformation but also generates important social benefits by improving population health. Future institutional reforms should therefore place greater emphasis on coordination between environmental and health policies. Improvements in population health should be incorporated into the comprehensive evaluation framework for environmental governance performance, thereby promoting the coordinated advancement of ecological governance and health protection.

Second, the pollution-reduction and environmental-governance incentives created by the environmental protection tax should be fully utilized. The empirical results suggest that reducing sulfur dioxide emissions and increasing investment in industrial pollution control may represent important channels through which the environmental protection tax improves population health. Accordingly, local governments should further strengthen coordination among tax collection and administration, pollutant-emission monitoring, and environmental law enforcement in the implementation of the environmental protection tax, thereby enhancing firms’ internal incentives to reduce emissions. At the same time, enterprises should be encouraged to transform the cost pressure generated by the environmental protection tax into long-term environmental investments, including the construction of pollution-control facilities, the adoption of cleaner production technologies, and the upgrading of production processes. Such measures would facilitate a shift in pollution control from end-of-pipe treatment toward source prevention and whole-process management, thereby more fully realizing the environmental benefits and health dividends of the environmental protection tax.

Third, differentiated environmental governance and health-promotion policies should be implemented in accordance with regional characteristics. The regional heterogeneity analysis indicates that the health-promoting effect of the environmental protection tax is relatively pronounced in the eastern and western regions, whereas its effect in the central region remains statistically uncertain. The eastern region should continue to leverage its advantages in economic development, green technologies, and environmental governance capacity and promote the coordinated implementation of the environmental protection tax and green industrial upgrading. The western region should further increase investment in environmental governance infrastructure and public services to fully realize the health benefits of pollution control. The central region should accelerate the transformation and upgrading of traditional industries, strengthen environmental tax administration and pollution-control capacity, and improve the effectiveness of environmental protection tax implementation.

Although this study provides a relatively systematic examination of the effect of the environmental protection tax policy on population health, several limitations remain and warrant further investigation. First, although the entropy weight method provides a more comprehensive measure of population health than any single indicator, it cannot fully capture all dimensions of health. Future research could incorporate additional micro-level health survey data or more detailed administrative health records to obtain a more refined measure of population health. Second, the analysis is conducted primarily at the provincial level, which limits its ability to capture variations in policy effects at more disaggregated geographical levels and prevents a deeper identification of the specific transmission mechanisms linking firm-level pollution reduction to improvements in population health. Future studies could use prefecture-level, county-level, firm-level, or individual survey data to investigate the micro-level mechanisms underlying the health effects of the environmental protection tax in greater depth. Third, this study focuses primarily on the average treatment effect of the environmental protection tax policy. Future research could combine spatial econometric methods with dynamic policy evaluation approaches to further examine the spatial spillover effects and long-term dynamic impacts of the environmental protection tax on population health.

## Data Availability

The original contributions presented in the study are included in the article/supplementary material, further inquiries can be directed to the corresponding author.
